# 1470 nm laser is better for prostate hyperplasia treatment with different volume size via transurethral enucleation

**DOI:** 10.1186/s12893-023-02266-2

**Published:** 2023-11-21

**Authors:** Zhou Fayou, Zheng Jiude, Zhang Shuxian, Shen Yajun, Xu Wei, Yu Jia, Su Fan, Xiong Yueling, Han Renrui, Tang Xiaolei

**Affiliations:** 1 Department of Urological Surgery, The Second Affiliated Hospital of Wannan Medical College, Wuhu, 241000 China; 2 Centre for Translational Medicine, The Second Affiliated Hospital of Wannan Medical College, Wuhu, 241000 China; 3 Vascular disease research center & Basic Medical Laboratory, The Second Affiliated Hospital of Wannan Medical College, Wuhu, 241000 China; 4 Research Office of Wannan Medical College, Wuhu, 241000 China

**Keywords:** Semiconductor laser, Benign Prostatic Hyperplasia, Enucleation, Therapeutic effect

## Abstract

**Introduction:**

The large amount of intraoperative bleeding and the high incidence of postoperative hematuria are still common factors affecting the prostate surgery treatment effect. Our research aimed to observe the effect of prostatic enucleation using 1,470 nm semiconductor laser on the amount of bleeding in patients with different sizes of prostate hyperplasia.

**Methods:**

According to the size of the prostate, forty eligible patients with benign prostatic hyperplasia (BPH) were enrolled and divided into low and high volume group in this study. Hemoglobin decline, urinating condition, complications and erectile function were collected and compared before and after surgery.

**Results:**

Our data showed that hemoglobin decline was (10.0 ± 6.2) g/L and (12.1 ± 7.8) g/L, respectively for two group after surgery (*P* = 0.363). Urination was significantly improved following surgery in both groups of patients (*P* < 0.05), and no permanent urinary incontinence and sexual dysfunction and so no serious complications occurred.

**Conclusion:**

The above results suggested that prostatic enucleation using 1,470 nm semiconductor laser can be safe and effective for prostatic hyperplasia, and this surgery produced no significant effect on the amount of bleeding in whatever size of the prostate.

## Introduction

Benign prostatic hyperplasia (BPH) is the most common disease in middle-aged and elderly men. The lower urinary tract symptoms caused by BPH seriously affect the quality of life of patients [1]. Surgical relief of obstruction is the most effective treatment for benign prostatic hyperplasia in the elderly [2]. With the application of laser technology in medical field, its application in prostatic hyperplasia surgery has gradually become the mainstream [1, 3], it’s good hemostatic function and tissue explosion can safely and effectively complete the resection and enucleation of prostate tissue [4, 5]. Clinical practice has proved that the application of laser technology can effectively improve the surgical efficacy and safety, where 1,470 nm semiconductor laser has vaporization efficiency high, good hemostatic performance and other good physical properties [6].

1470 nm laser vaporization efficiency is high, the depth of tissue penetration is moderate, and the hemostatic effect is good, which can effectively reduce the risk of bleeding, and reduce the operation time, bladder flushing time, indwelling catheter time and hospital stay [7]. It has been widely used in prostatic augmentation surgery. From July to December 2019, 40 cases of benign prostatic hyperplasia were performed by 1470 nm semiconductor laser prostatic enucleation. The results are reported as follows.

## Materials and methods

### Clinical data

Forty patients with BPH admitted to the Second Affiliated Hospital of Wannan Medical College from July to December 2019, aged 52 ~ 87 years, with prostate volume of 32 ~ 142 mL and disease course of 2 ~ 10 years, were selected. Inclusion criteria: (1) Drug-ineffective with residual urine > 50 ml (2) History of acute urinary retention (3) Repeated urinary tract infections combined with bladder stones (4) Complicated renal impairment or inguinal hernia (5) Can tolerate surgery. Patients with BPH were divided into two groups according to prostate volume, 20 patients with small and medium BPH < 60 mL as the observation group, and 20 patients with BPH ≥ 60 mL as the control group. Neurogenic bladder and prostate cancer were excluded before surgery. For oral anticoagulant drugs such as aspirin, the drug should be stopped for more than one week, and surgery should be arranged after coagulation function is normal. Patients with chronic diseases such as cardiopulmonary diseases should be actively treated before surgery to meet the surgical standards. Comparison of preoperative baseline indicators between the two groups (Table 1).

**Table 1:** Comparison of preoperative coagulation function indexes between the two groups (n = 20, ‾x ± s)

Group	Age	PV/mL	PT/s	PTR	PT-INR	FBG/(g/L)	APTT/s	TT/s
Low volume group	72. 0 ± 6.0	45.5 ± 9.3	10. 6 ± 0.7	0.9 ± 0.1	0. 9 ± 0.1	2.9 ± 1.0	27.0 ± 3.1	18. 0 ± l.3
High volume group	71. 5 ± 8.7	87. 3 ± 19.9	10. 6 ± 1.2	0.9 ± 0.1	0.9 ± 0.1	2.7 ± 1.1	27.6 ± 6.8	17. 7 ± 1.4
*t*	0.238	8.510	0.113	0.039	0.248	0.566	0.342	0.606
*P*	0.815	0.000	0.910	0.969	0.805	0.575	0.754	0.548

Note: PV:Prostate volume; PT:Prothrombin time; PTR:Prothrombin time ratio; PT-INR: Prothrombin time-International standardization ratio; FBG:Plasma fibrinogen; APTT: Partial thromboplastin time; TT: Thrombin time

### Instruments and equipment

1470 nm semiconductor laser was used in the surgical treatment system, with vaporization power of 30 ~ 150 W and electric coagulation power of 20 ~ 50 W. 26 F reflux cystoscope produced by Germany Storz Company was used. YSB - III Medical surgical comminutor manufactured by Hangzhou Hawk Company.

### Surgical methods

The surgical methods of the two groups were the same, were treated with 1470 nm laser prostatic enucleation. General anesthesia or lumbar anesthesia, lithotomy position, using direct transmission fiber, diameter 600 μm, the perfusion fluid as the physiological saline, The position of verumontanum was determined as anatomic mark during the operation, the vaporization power was set to 140 W and the electrocoagulation power to 40 W. The higher the power, the stronger the tissue vaporization or coagulation effect. According to clinical experience, the vaporization power is set to 140 W and the electrocoagulation power is 40 W, which can meet the needs of the operation. Firstly, the prostate tissue was vaporized longitudinally along the bladder neck at 5 and 7 points respectively to the anterior edge of the verumontanum, forming a groove with a depth close to the surgical capsule. Then, the prostate mucosa was vaporized laterally to approach the capsule at 0.5 cm at the leading edge of the verumontanum. The tip of the endoscopic body was used to swing horizontally from the shallow groove at the leading edge of the verumontanum to pry the gland and expose the surgical capsule. The middle gland was removed by retrograde blunt push, and the middle gland was removed. The prostate tissue was vaporized to the surgical capsule between 1 and 11 point at the top of the bladder neck, and stopped at about 1 cm in front of the urethral extender muscle to avoid the injury of the external bladder sphincter. The glands on both sides of the bladder neck were vaporized from top to bottom from 1 point or 11 point to the capsule layer successively, reaching 3 point on the left side and 10 point on the right side. After vaporizing and cutting the glands on the parietal lobe and the upper half of the left and right lobes, the residual glands on the lower part of the left and right lobes were enucleation. Complete hemostasis of the surgical wound, the bladder was filled with the perfusion solution, and the tissue was crushed and sucked out of the body. No residual glands and injuries were detected in the bladder, and the F20 three-chamber balloon catheter was indwelled and irrigated continuously with normal saline.

### Observation indicators

Surgical time, prostate resection quality(The wet weight mass of prostate gland tissue after surgical resection in two group), postoperative bladder flushing time, indwelling catheter time, hospital stays, urination and complications were recorded. The scores of International Erectile Function five Scale (IIEF-5) [8] were used to evaluate patients’ sexual function from everybody after hospital discharge for six months.

### Statistical analysis

SPSS 18.0 statistical software was used to analyze the datum in this study. The measurement data was expressed as means ± standard deviation (s.d.), two independent sample *t*-test and paired *t*-test were used for comparison difference between groups. *P* < 0. 05 was considered statistically significant. All of the data have been standardized.

## Results

### Comparison of surgical conditions between the two groups

The operation time of observation group was shorter than that of control group (*P* < 0.05), and The quality of prostate resection was less than that of control group (*P* < 0.05). However, there were no significant differences in Bladder irrigation time, indwelling catheter time and postoperative hospital stays between 2 groups (*P* > 0.05). (Table 2).

**Table 2:** Comparison of surgical conditions between the two groups (n = 20, ‾x ± s)

	operation time/min	Prostate mass/g	Bladder flushing time/h	Time of indwelling catheter/d	Postoperative hospital stay/d
Low volume group	52. 9 ± 10.8	24. 8 ± 6. 0	15. 4 ± 2.8	4. 5 ± 0. 5	5. 7 ± 1.0
High volume group	74. 0 ± 13.7	52. 7 ± 16.7	16. 3 ± 2.5	4. 6 ± 0. 5	5. 9 ± 1.0
*t*	5.393	7.021	1.059	0.620	0.650
*P*	0.000	0.000	0.296	0.539	0.520

### Comparison of RBC, HGB, HCT and other indicators between the two groups before and after surgery

As shown in Table 3, there were no significant differences in RBC, HGB, HCT and other indicators between 2 groups before treatment (*P* > 0. 05). in observation group, HCT, RBC and HGB decreased after treatment compared with before treatment (*P* < 0. 05), while MCV, MCH and MCHC had no statistical significance before and after treatment (*P* > 0. 05). The RBC and HGB of the control group after treatment were better than those after treatment, there was no significant difference in HCT, MCV, MCH and MCHC before and after treatment (*P* > 0.05). However, there was no significant difference between 2 groups before and after treatment (*P* > 0. 05).

**Table 3:** Comparison of RBC, HGB, HCT and other indicators between the two groups before and after surgery (n = 20, ‾x ± s)

		HCT/%	MCV/fL	MCH /pg	MCHC/(g/L)	RBC/(10^12/L)	HGB/(g/L)
Low volume group	preoperative	39.8 ± 4.5	91.3 ± 4.1	30.3 ± 1.4	331.4 ± 10.3	4.3 ± 0.5	132.5 ± 17.4
	postoperative	35.4 ± 4.4	91.5 ± 3.7	30.2 ± 1.2	330.6 ± 7.7	3.9 ± 0.5	122.5 ± 14.0
	$\bar {\rm d}$±s_*d*_	4.4 ± 3.6	-0.2 ± 1.4	0.l ± 0.8	0.9 ± 9.8	0.5 ± 0.4	10.0 ± 6.2
*t*		5.492	0.633	0.323	0.389	5.783	7.265
*P*		0.000*	0.554	0.751	0.701	0.000*	0.000*
High volume group	preoperative	38.9 ± 7.0	93.8 ± 4.5	30.9 ± 1.7	329.1 ± 11.2	4.4 ± 0.5	132.7 ± 17.3
	postoperative	36.6 ± 4.8	93.4 ± 4.3	30.9 ± 1.9	331.0 ± 10.7	3.7 ± 0.7	120.7 ± 16.7
	$\bar {\rm d}$±s_*d*_	2.3 ± 7.3	0.4 ± 1.3	-0.l ± 0.8	-1.9 ± 9.9	0.7 ± 0.4	12.1 ± 7.8
t		1.400	1.258	0.411	0.859	7.618	6.882
*P*		0.178	0.224	0.686	0.401	0.000*	0.000*
t*		1.153	1.308	0.515	0.885	1.883	0.920
*P*		0.256	0.199	0.609	0.382	0.067	0.363

Note: HCT: Red blood cell specific volume; MCV: Mean corpuscular volume; MCH: Mean corpuscular hemoglobin; MCHC: Mean hemoglobin concentration of red blood cells; RBC: Red blood cell; HGB: Hemoglobin. * represents the difference *t* test results of two groups

### Comparison of urination before and after treatment between the two groups

The results (Table 4) showed that there were no significant differences in residual urine volume (RUV), IPSS, Qmax and QOL before treatment between 2 groups (*P* > 0. 05). After treatment, RUV, IPSS and QOL in 2 groups were lower than before treatment (*P* < 0. 05), while Qmax was higher than before treatment (*P* < 0. 05). However, there was no significant difference between 2 groups before and after treatment (*P* > 0. 05).

**Table 4:** Comparison of urination before and after treatment between the two groups (n = 20, ‾x ± s)

		RUV	IPSS	Qmax	QOL
Low volume group	preoperative	33.0 ± 36.3	20.6 ± 2.3	8.5 ± 1.3	4. 5 ± 0.6
	postoperative	6.6 ± 7.8	9.5 ± 2.5	19. 8 ± 2.4	1. 9 ± 0.7
		26.4 ± 30.3	11.1 ± 2.4	-11.3 ± 2.8	2.7 ± 1.2
*t*		3.885	20.829	18.822	10.025
*P*		0.000*	0.000*	0.000*	0.000
High volume group	preoperative	84.2 ± 118.0	21.4 ± 1.9	7.9 ± 1.7	4.8 ± 0.7
	postoperative	13.8 ± 15.0	11.3 ± 2.6	18.8 ± 2.4	1.7 ± 0.6
		70.4 ± 105.2	10. 1 ± 2.8	-10.9 ± 3.0	3.2 ± 0.2
*t*		2.993	16.419	16.403	11.917
*P*		0.007*	0.000*	0.000*	0.000*
t*		1.799	1.169	0.392	1.338
*P*		0.080	0.249	0.698	0.189

Note: RUV: Residual urine volume; IPSS: International Prostate Symptom Score; Qmax:Maximum urinary flow rate; QOL: Quality of life score* represents the difference *t* test results of two groups

### Comparison of erectile function between the two groups before and after with different treatments

All the patients donors, age below 70 years old, were selected during follow-up for sexual function questionnaire using IIEF-5. The questionnaire contains 5 dimensions about erectile function and all dimensions were divided into five grades, scored from 1 to 5. According to the international standard, the score of less than 7 is major erectile dysfunction, 8 to 11 is moderate erectile dysfunction, and 12 to 21 is mild erectile dysfunction. In this study, both groups’ patients showed mild erectile dysfunction before surgery, and there was no significant difference in scores (*P* > 0. 05). Although these patients still had some mild erectile dysfunction after surgery, the average score of both groups were higher than before, and the difference was statistically significant (*P* < 0. 05). (Table 5).

**Table 5:** Comparison of erectile function between the two groups before and after with different treatments (‾x ± s)

	Observation group (n = 12)	Control group (n = 14)	*t*	*P*
preoperative	15.53 ± 3.93	14.95 ± 4.24	0.3595	0.7223
postoperative	18.24 ± 2.11	18.09 ± 3.58	0.1272	0.8998
*t*	2.105	2.117	—	—
*P*	0.047*	0.044*	—	—

Note: The results in the table are scores of IIEF-5.Postoperative data were obtained at 6 months. * represents the p < 0.05; ** represents the p < 0.01

### Incidence of Complications after treatment in the two groups

There were 2 cases of temporary urinary incontinence in the observation group and 3 cases of temporary urinary incontinence in the control group. After levator ani muscle training, all of them recovered within one week, and no massive bleeding or permanent urinary incontinence and sexual dysfunction occurred in the two groups.

## Discussion

At present, laser technology has been widely used in the operation of prostate hyperplasia, in which 1470 nm semiconductor laser can be absorbed by both water and hemoglobin [7, 9]. The thickness of coagulation layer produced during the operation is about 0.4 ~ 0.6 mm, which has good tissue ablation and hemostasis effect [10]. The operation is not limited by the size of the gland and has broad application prospects.

Bleeding and urinary incontinence are common complications of transurethral enucleation of the prostate [11]. Transurethral plasma prostatic enucleation has the advantages of short operative time and few remaining glands [12], but compared with 1470 nm laser, relatively more intraoperative bleeding is its disadvantage, and the incidence of postoperative temporary urinary incontinence is as high as 22.4% ~ 43.1% [13]. Holmium laser can enucleate the gland completely between the capsule and the gland with good tissue microblasting function, and the effect is definite, but the incidence of postoperative temporary urinary incontinence is high. [14] After 1 month, 22 patients (31.9%) developed stress incontinence, and after 3 months, 6 patients (8.6%) developed stress incontinence. The green laser is only absorbed by hemoglobin, and the coagulation layer generated on the intraoperative prostate wound is 1.5 nm thick. Postoperative eschar loss can cause delayed bleeding and severe urinary tract irritation [15]. Because 1470 nm semiconductor laser has the advantages of double absorption in water and hemoglobin, it can quickly solidified and seal most small blood vessels in the process of tissue vaporization cutting, and the hemostatic effect is better than similar wavelength laser [16, 17]. In this study, blood routine tests were performed on the second day after 1,470 nm laser enucleation of prostate. Compared with preoperative blood routine tests, the amount of surgical hemoglobin loss was calculated. The results showed that the average amount of hemoglobin loss in the observation group was (10.0 ± 6.2) g /L. The average loss of hemoglobin in the control group was (12.1 ± 7. 8) g /L, and there was no significant difference between the two groups (*P* = 0. 363), It was confirmed that 1,470 nm laser enucleation of prostate had no significant effect on the intraoperative blood loss of different prostate volumes. This is similar to the findings of the Jun Zhang study [18],

The findings presented here are similar to those of a randomized controlled trial [19], The patients treated with 1470 nm diode laser enucleation of the prostate had significantly decreased operation time (74.6 ± 17.0 vs. 98.8 ± 18.9 min, p < 0.001), hemoglobin loss (1.06 ± 0.49 vs. 1.59 ± 0.60 g/dL, p < 0.001), bladder irrigation time (22.1 ± 8.1 vs. 33.9 ± 10.0 h, p < 0.001), catheter duration (3.2 ± 1.3 vs. 5.8 ± 1.0 days, p < 0.001), and hospital stay (7.6 ± 1.4 vs. 9.6 ± 1.3 days, p < 0.001) compared with the plasmakinetic resection of the prostate group.

The common causes of temporary urinary incontinence are fatigue injury or rupture of urethral sphincter caused by the traction and compression of urethral sphincter caused by the lens sheath during prostatic enucleation [20]. After enucleation, the prostate fossa cavity was larger and the posterior urethral resistance decreased significantly in a short time. The external urethral sphincter at 10 o ‘clock to 2 o ‘clock during the dissection of the gland is prone to tear injury when the gland is enucleated [21, 22].

Postoperative prostate fossa infection, although postoperative measures such as levator ani exercise can restore normal urinary control, but still bring confusion and anxiety to patients [23]. In order to reduce the occurrence of postoperative temporary urinary incontinence, it is important to reduce the prying action during enucleation [24, 25].

During the operation, the combined method of enucleation and vaporization was adopted in this study to minimize the prying force, rationally utilize the vaporization function, effectively reduce the risk of sphincter injury, and achieve the purpose of rapid hemostasis. According to the mechanical characteristics [26], when the glands at 2 or 10 point were removed, the external sphincter as the fulcrum exerted the greatest compressive force, and the external sphincter was easily damaged at this time [27]. In this area, we adopted the method of vaporization cutting, that is, after the parietal tissue vaporized between 1 point and 11 point. The two lobes were vaporized from the bladder neck at 1 and 11 points to the capsule level from top to bottom, left to near 3 point, right to near 9 point. It was relatively easy to enucleate the rest of the glands. In case of nodules or adhesion, rapid vaporization was performed to avoid forcible prying force.

Erectile function was not negatively affected but partially improved after laser prostate surgery. In view of the analysis of this situation, the original patient urination difficulty or retention of urine through surgery to relieve the patient’s body pain relieved the psychological burden, good mood will also lead to better sexual function [28, 29]. In addition, the distance between sexual nerve and prostate capsule is about 3 ~ 5 mm, 1470 laser energy will not cause thermal damage to sexual nerve [30, 31] This may also be one reason why the method improves sexual function.

In conclusion, as a result of 1470 nm semiconductor laser in the physical properties of water and double absorption in hemoglobin, make it have efficient tissue ablation and exact hemostatic function, using intraoperative enucleation and vaporization cutting method of combining, effectively reduce the risk of bleeding and urinary incontinence, has obvious advantages in terms of increasing the safety of operation.

The limitations of this study is based on data obtained in the sixth postoperative month. So juxtaposing these results with the short-term results of HoLEP to lack homogeneity.

## Conclusions

1470 nm semiconductor laser as a method for prostate hyperplasia treatment has the advantages of short operation time, small amount of blood loss, less complications, fast postoperative recovery, little impact on sexual function, and more conducive to improving the quality of life of patients.

## Data Availability

The datasets generated and analyzed during the current study are not publicly available due to patient privacy but are available from the corresponding author on reasonable request.

## Abbreviations

BPH: Benign prostatic hyperplasia
IIEF-5: International Erectile Function five Scale
RUV: Residual urine volume
